# The Lymphatic System, Considered in Relation to Its Anatomy, Physiology, and Pathology

**Published:** 1837-10

**Authors:** 


### Art. II.

*Le Système Lymphatique, considéré sous les Rapports Anatomique, Physiologique, et Pathologique.* Par G. Breschet.—*Paris*, 1836. 8vo. pp. 304.

*The Lymphatic System, considered in Relation to its Anatomy, Physiology, and Pathology.* By G. Breschet.—*Paris*, 1836. With four Plates.

The present work, like most of M. Breschet's former ones, is calculated to inspire us, if not with a very high notion of his originality, at least with respect for his industry and patience in the collection of facts, as well as for his judgment in comparing and disposing of the theories of others. When we consider that it was got up under all the hurry and agitation attendant on the "Concours," (as appears from page 51,) its merits, whatever they may be, are in a manner enhanced; and, whatever defects it displays should meet with more than ordinary critical indulgence.

In the commencement of his work, M. Breschet gives us a history of his subject, beginning with the conjectures of Aristotle and the demonstrations of Erasistratus and Herophilus, and ending with the recent investigations of Lauth in Belgium and of Panizza in Italy.

Galen, who ascribed the office of intestinal absorption to the mesenteric veins, seems to have been quite ignorant of the uses and importance of the system under consideration; and his authority reigned so absolute for many centuries after, that not only his positive but even his negative opinions, or his very silence, were implicitly respected; so that, as late as the year 1565, the distinguished Italian anatomist, Eustachius, though he had actually discovered the main trunk of this set of vessels, dared not, in opposition to the above authority, heretically to believe his own eyes, but concluded it, in spite of all evidence to the contrary, to be a mere vein. Aselli, in 1622, detected both the lacteals and the lymphatics of the liver in dogs and various other animals, without, however, being led to a knowledge of their true nature; for such is the force of prejudice and the tyranny of long-established opinion, that he saw nothing in the discovery but a corroboration of Galen's theory of the sanguification of the vital fluid in the liver, conceiving that the vessels in question were running *towards* this organ, instead of passing out from it, as is really the case. In 1628, the lymphatics of the mesentery were seen for the first time in the human subject; but there was still much opposition to their being considered as a distinct system of vessels; and it is painful to think that the high names of Gassendi, Riolanus, and even of our own Harvey, who had himself suffered so grievously from the undue authority of the ancients and from the obstinate prejudice of his contemporaries, are to be found in the list of opponents. In 1649, Pecquet, a provincial physician in France, discovered anew the thoracic duct, which had been forgotten since Eustachius's time, and demonstrated it to be the common trunk of the system. Prejudice and obstinacy were at length obliged to yield to the force of truth; and it was clearly made out by Glisson and Vessling, by referring to the position of the valves, that the colourless vessels between the liver and mesentery really passed from the former to the latter, on their way to the thoracic duct.

The existence of the general system of lymphatics coming from all parts of the body had, up to this period, been quite unknown. The honour of the discovery was now disputed by Rudbeck, Bartholin, and Jolyff; but the Swede seems to have a fairer title to it than either the Dane or the Englishman. It was in 1651 that Rudbeck first saw them, and in 1652 he demonstrated them publicly before Queen Christina and others. The details of the subject were afterwards followed up with great zeal by Nuck, Ridley, Ruysch, Albinus, Meckel, and Lieberkuhn; by the Hunters, Hewson, Monro, Cruikshank, and Sheldon; by Soemmerring, Schreger, Werner, Feller, Haase, and Mascagni. By some of these it was carefully investigated in relation to its comparative anatomy; and in this department considerable accessions to our knowledge have still more recently been made by Fohmann, Lauth, Rossi, and Panizza; yet withal the precise mode of origin of these vessels has not hitherto been satisfactorily made out. Such is their tenuity, that the sense of sight, with all the aid that microscopes and skilful injections have furnished it, has never yet been enabled to detect how their minute ramifications commence in the various tissues of the body. But, as usual, where the senses fail us, there has not been wanting an abundance of fanciful hypothesis. Aselli, who was one of the first who endeavoured to ascertain this point with any degree of accuracy in regard to the lacteals, came to the conclusion that they commence by absorbent pores opening on the mucous surface; and this is the opinion which has been most generally held since his day. Indeed, the lymphatics of the whole body have been supposed to commence in a similar manner, from the various serous cavities, the cellular membrane, the surfaces of the arterial and venous tubes, excretory ducts, &c. Some anatomists, however, observing that the finer injections passed from the arteries into the lymphatics, thought themselves justified in concluding that the latter communicated freely with the former, or originated by inosculations with their minuter ramifications. This view of the matter has been supported by Cowper, Senac, Cheselden, and other high authorities. On the other hand, Panizza, one of the most recent investigators, has repeatedly injected the arteries of the intestines in the human subject, as well as in the horse, birds, the tortoise, &c., yet has never seen the fluid employed appear in the lymphatics. In the dog, on the contrary, on injecting the arteries of various portions of the intestinal canal, and more especially of the rectum, where the lymphatics are very numerous, he has frequently observed that these latter vessels became filled immediately after the entry of the fluid into the arterial capillaries; and this without the least appearance of extravasation. He has noticed a similar fact in relation to the small intestines of the hog; and, in injecting the hepatic artery in the human subject with mercury, the neighbouring lymphatics are almost always filled, and that too even where the fluid does not enter either the vena cava, vena portæ, or the biliary ducts. The same observation holds good in relation to the liver of the dog and of the horse, but fails in regard to reptiles. The injection of the splenic artery in man, in the dog, and in the hog, does not fill the lymphatics of the spleen; whilst, in the horse, some of these vessels on the convex surface of this organ, occasionally become thus filled. In man, and the mammifera generally, and in birds, the fluids injected into the renal artery do not enter the lymphatics of the kidney. Such are some of the singular anomalies which the experiments of M. Panizza have made known to us.

M. Breschet, in his researches in regard to the venous system, has observed that the lymphatics more frequently become filled when several veins are simultaneously injected.

M. Panizza asserts that no direct union nor continuity between the venous capillaries and the lymphatics has ever been made manifest to the eye, either in the human subject or in the lower animals, and affirms that the mode of mutual relation between the ultimate ramifications of these two systems is still one of nature's secrets; and consequently he refuses his assent to M. Fohmann's opinion as to the inosculation of their capillaries; as this, as well as the other view, according to which each of these two sets of vessels is supposed to terminate by open orifices, is a mere hypothesis. He has himself always observed that, in those cases where the injection passed from the sanguiferous vessels into the lymphatics, a network of microscopic capillaries surrounded the ramifications of the latter, and conceives that the penetration of the fluid may be ascribed to porosity.

Mascagni long since alluded to the facility with which we may mistake the veins coming from the glands, especially in the abdomen, for lymphatic trunks inosculating with the neighbouring veins. This error may be avoided, according to Panizza, by attending to the following circumstances:—the venous ramifications are much more rectilinear in their direction than the lymphatics, which are comparatively tortuous; they are more cylindrical and less knotted, from the absence of valves in the smaller veins, and we can consequently easily make the fluid retrograde in them towards the gland; their walls are thicker; the fluid which they contain is red; and, lastly, their mode of inosculation is different from that of the lymphatics.

M. Panizza points out two peculiar circumstances under which, if great caution be not employed, we may still be led to confound a vein with a lymphatic; namely, first, when a vein returning the blood from a gland lies concealed by several lymphatic efferent vessels, which accompany it to the next large vein; the appearance of mercury in the large vein, on injecting the part, naturally enough gives rise to the erroneous conclusion that the fluid had made its way thither through the lymphatics, which are alone seen, and that they open directly into such vein; whereas, it has really been carried by the vessel which lies hid in the midst of them;—secondly, where a small vein, coming out from a gland, opens after a short passage into a larger one, the mercury passing freely from the former into the latter, the short vein may, from its situation, be readily mistaken for the lymphatic efferent of the gland. But the error is easily detected by tying the vein; upon which the fluid, being now arrested in its former course, forces its way into the real efferent, and the difference between the two vessels becomes immediately obvious.

It was the opinion of Mascagni that lymphatics existed in every tissue in the body; but the more general belief was, that, though they were to be detected in most of the organs, yet the brain, the spinal marrow, the eye, and the placenta were unprovided with them. M. Fohmann, however, has, within these four years, succeeded in injecting them on the surface of the brain, in the substance of its membranes, and, as he believes, also in the umbilical cord and on the placenta. M. Arnold thought he had detected them in several of the tissues of the eye; and Sir Everard Home, in the minute canal which traverses the retina in the middle of the yellow spot of Soemmerring, saw only a lymphatic vessel. Breschet puts little faith in either of these two latter assertions.

M. Fohmann, as we have just stated, has described and represented lymphatic vessels in the umbilical cord and in the placenta. Long since similar observations had been made by Needham, Pascoli, Wrisberg, and others; but these had fallen into disrepute, inasmuch as they were not confirmed by the subsequent investigations of Hunter, Hewson, Cruikshank, and Mascagni. The umbilical cord is conceived by M. Fohmann to consist almost entirely, with the exception of its sanguiferous vessels, of a plexus of absorbents, so that one can scarcely introduce the point of a needle without touching some of them; and to fill them, it is only necessary to pierce the sheath of the funis with a finely pointed lancet, and then to introduce mercury by means of a small tube and very moderate pressure. He has not found any valves in these vessels: indeed, the difference of their general appearance from that which lymphatics present in other parts, is sufficient to account for the doubts expressed in regard to them by Panizza and others. M. Fohmann himself admits that he has never seen any vessel of this kind passing from the cord to ramify on the amnion, and rarely any such penetrating into the parenchyma of the placenta. M. Breschet says that he also has succeeded in injecting these supposed lymphatics of the umbilical cord, both directly in the manner specified above, and also indirectly through the lymphatics of the liver.

Professor Lauth thinks he has detected lymphatics between the decidua and the uterus; a discovery received with much doubt by Carus, and which Breschet conceives to stand in need of further confirmation. Lauth's notion of the matter is, that, there being no direct communication between the uterine vessels and those of the placenta,—no cells into which the blood, as fancied by some, is poured out,—the only connexion of the maternal with the fœtal circulation is through the medium of two sets of lymphatics; one of which he supposes to terminate in the sanguiferous vessels of the placenta, and the other in those of the decidua and uterus; that by the one the blood (or at least the materials for its formation,) is transferred from the mother to the fœtus; and by the other the constituent parts of the effete blood of the fœtus are taken back again into the mother's system. M. Breschet admits the ingenuity of this theory, whilst at the same time he feels obliged to reject it, as being altogether deficient in anatomical proof. Indeed, M. Lauth himself, in consequence of his own more recent investigations, has been led to doubt the accuracy of his former observations; and he has had the courage and good faith to acknowledge this change of opinion.

The cellular tissue throughout the body seems to be the source from which the lymphatics almost exclusively take their origin; and it is accordingly in those parts where it is wanting, such as the nails, epidermis, hair, enamel of the teeth, &c., that these vessels seem entirely absent.

Cruveilhier thinks it probable that the cellular tissue and serous membranes are made up of lymphatics; and Mascagni, by one sweeping and, as it seems to us, somewhat extravagant generalization, lays it down that all the white tissues are thus constituted. Professor Arnold, of Zurich, with microscopes magnifying to thirty times and upwards, observed that the cellular tissue at the back of the eye consisted almost entirely of a network of lymphatics, in the meshes of which lies the fat forming the supporting cushion to the organ; and M. Fohmann, by his injections, has been led to a similar conclusion. Müller, of Berlin, has, however, thrown some doubt on the correctness of these observations; and Breschet likewise thinks they require at least to be substantiated.

M. Lauth, in injecting the lymphatics of the leg of an anasarcous subject, succeeded in filling the minute cutaneous ramifications in the region of the groin and inside of the thigh so completely, that it was scarcely possible to put down the point of a needle without touching some of them; and the same anatomist, as well as Tiedemann, Cruveilhier, and Breschet himself, has been equally successful by another method,—namely, by just penetrating the cutaneous surface with a capillary tube of steel or glass, containing mercury, in such a manner as to implicate only the epidermis, and reach the vascular network between it and the chorion.

These minute ramifications are without any complete valves, but present here and there obvious dilatations, especially at the points of junction of the leading branches of the network. M. Breschet observes, that these dilatations are less frequently met with on the surface of the true skin than within its substance. He remarks likewise that this network sends out into the substance of the epidermis certain minute, loop-like prolongations or doublings of the lymphatic vessels; but that he has never been able to discover that they terminate by any open orifice or free extremity. He also notices that the network of lymphatics is more superficial than that formed by the sanguiferous capillaries: the two are, however, very intimately blended, more especially within the substance of the true skin. The preceding observations are only confirmatory of similar ones already made by M. Fohmann. But neither Breschet, Fohmann, nor Lauth have ever succeeded in forcing the mercury in the lymphatics through the surface of the cuticle; and hence they conceive that, if Haase did so, it must have been by using such violence as to lead to their rupture. Breschet looks upon this non-escape of the fluid as an additional proof that these vessels are unprovided with any aperture at their extremity; and this is likewise the opinion of Panizza and Fohmann. Indeed, it has been found by one of them that we may remove the thin epidermis of the glans penis, the part having been previously minutely injected with mercury, and yet none of this fluid shall escape or adhere to the membrane so removed; which seems obviously to imply the integrity of the lymphatic vessels after such removal, and that they do not penetrate through its surface, nor possess any kind of terminal aperture.

In the mucous membrane, the loop-like terminations of these vessels lie almost naked, or simply enveloped in mucus and very loose cellular tissue, which is just sufficient to keep them in connexion with the sanguiferous capillaries; a state of things, as Breschet adds, eminently favorable to the act of absorption. The villosities are, in fact, composed of loops of these vessels and of the arterial and venous capillaries, and terminal apertures are no more discoverable in the lymphatics here than in those of the skin already spoken of. As to this point, however, Breschet, Fohmann, and Panizza stand in opposition to Cruveilhier and Magendie, who believe in the existence of open orifices on the extremities of the villi.

The condition of these vessels on the surface of other mucous membranes, excretory ducts, &c., varies little from that just described, save as to slight differences in regard to tenuity, number, &c., detailed at length by M. Fohmann, to whose work we refer the reader.*

Many anatomists conceive that the lymphatics take their rise, amongst other situations, from both the inner and the outer walls of the blood-vessels. This, it is obvious, is quite a distinct matter from their being continuous with these latter vessels, which they undoubtedly are not; else they would always be susceptible of being injected by filling the arteries or veins, which, as we have already seen, is very far from being invariably the case; and in those cases where such passage from the one to the other system of vessels actually occurs, it has been supposed by Monro, Meckel, Caldani, Mascagni, and Béclard that the injected fluid previously undergoes an extravasation into the cellular membrane. M. Panizza has, however, observed it without any such extravasation; which indeed, at best, seems to be but an unsatisfactory explanation of the occurrence. Breschet thinks we must rest satisfied with the admission of the fact, and that we are not yet in possession of any adequate explanation of it. M. Fohmann has succeeded in injecting minute networks of lymphatics on the inner surface of the arteries, and has represented them in his plates. He has also demonstrated their existence in the interior of the veins, but their injection is here much more difficult. He has succeeded in injecting them also in the inner as well as the outer lining of the heart; as have likewise MM. Cruveilhier and Bonamy. Fohmann has, moreover, demonstrated their trunks within the skull on the anfractuosities of the brain following the course of the veins. There is no organ in which they are more difficult to inject than in the muscles, owing to the extreme tenuity of their coats in this situation. The author last named has succeeded in respect to the diaphragm alone, where they are seen to follow the bundles of muscular fibres in their course, and form meshes around them, and appear to be destitute of valves.

The lymphatics of the nerves, though their existence was pretty generally admitted, had never been satisfactorily demonstrated till M. Fohmann turned his attention particularly to the subject, and fully succeeded in injecting them.

It has commonly been supposed that the brain is ill supplied with lymphatic vessels. This, as we have just seen, is certainly not true in regard to its membranous envelopes. According to the author just alluded to, if we introduce a lancet between the pia mater and the arachnoid, and inflate the artificial canal so made, a network of lymphatics is immediately rendered obvious between these membranes, the branches of which are larger than in other parts of the body, whilst their parietes

* Mémoire sur les Vaisseux Lymphatiques de la Peau, les Membranes Muqueuses, &c.—Liege, 1833.

are so feeble as to tear on every attempt to introduce mercury into them. In their ramifications they accompany, as already stated, the arteries and veins. The old account which Ruysch has given of them, and of their difference from those of other tissues, is better than that which Mascagni has more recently furnished us with. The existence of these vessels in bone has been fully demonstrated by the successful injections of Cruikshank, Soemmerring, and Bonamy.

The dimensions of the lymphatics differ considerably in the different tissues, but, according to M. Müller, one of the latest authorities on the subject, are never so small as the arterial and venous capillaries, and are always, without exception, visible to the naked eye. M. G. R. Treviranus, in a work which has just appeared, says that he has discovered by the microscope that the walls of the lymphatics, like the cellular membrane and other tissues, are made up of minute elementary cylinders, of a diameter of from about 0.001 millimetres to 0.006, placed in a series side by side and end to end, so as to constitute tubes which form networks, and open into larger lymphatic trunks. According to the same authority, they originate also in the villi; and their roots in this situation are just the elementary cylinders of the cellular tissue, which unite together and terminate in the extremity of some larger lymphatic. If we take an animal which has been fed shortly before death, and examine with a powerful microscope the villi strongly illuminated from below, we may observe, he asserts, on their surface prominent vesicles, from the sides of which there descend obscure lines, which unite in a point in the axis of each of the villi. These, Treviranus believes, can be nothing but the roots of the lacteals; as their straight direction and central position sufficiently distinguish them from the sanguiferous capillaries which run along the surface of the villi, and are more sinuous in their outline. He goes on to state that, on the surface of each of the prominent vesicles, a circular point is discoverable, which is constant, and very distinguishable from other smaller points visible in the same part; and he coincides with Cruikshank in supposing it to be an aperture and the commencement of the lacteals. He adds, however, what tends rather to shake our faith in the correctness of his conclusion, that it might also be considered as a microscopic mucous follicle seated on each of the villi, and that it may possibly unite both of these very dissimilar functions. Moreover, his observations having been made with very high magnifying powers, are not wholly exempt from a portion of that doubt which seems almost inseparable from results so obtained. M. Breschet is still of opinion that these apertures have not been yet sufficiently demonstrated, and that the origin of the lymphatics are so minute, and the fluid which they convey so transparent, that the mode in which they commence is still a secret. Their valves, by preventing in a great degree the retrograde motion of artificially injected fluids, present an additional difficulty in the way of the investigation.

The third section of M. Breschet's work, "On the General Arrangement of the Lymphatic System," contains scarcely any thing which can be called new. He begins by mentioning the arrangement of the minuter lymphatics into fine networks, from which branches of a larger size take their rise; by these, still larger meshes are produced. The direction of the trunks originating herefrom approaches to rectilinear; they gradually converge towards the main trunk of the system, forming in their progress two planes in relation to the respective organs in which they originate; a superficial one immediately beneath the external envelope of the organ, —(in the skin, for example, they accompany the subcutaneous veins;) —and a deep-seated one accompanying the arteries and nerves of the part. The deep-seated ones are larger than the superficial; those of the inferior extremities larger than those of the superior; whilst those of the head, on the contrary, are remarkably small. He alludes also to the fact of their changing their dimensions with changes in the condition of the organ from which they rise: thus, they become enlarged in the gravid uterus, in the breasts of women giving suck, in the case of suppuration and scirrhus of a part.

Their universal mode of origin seems to be by delicate plexuses, save in the single instance of the villi, in which, as we have seen, they commence in the form of loops. As they ordinarily run parallel, and do not converge or become lost in one another like veins, their chief mode of communication is by means of cross branches, and they often run a considerable distance without any change in their dimensions. They are susceptible of a very high degree of dilatation, and are, for the most part, capable of sustaining a much higher column of mercury without rupture than either arteries or veins of a similar caliber. On this latter point the experiments of Sheldon and Meckel are in perfect accordance with those of Werner and Feller.

As to their structure, the commonly received opinion is that their walls consist of two membranes—an external cellular one, of considerable density and firmness and very elastic; and an internal one, finer, more delicate, and more extensible than that of the veins, and by its folds forming the valves. They have no middle fibrous membrane, like the arteries. Schreger, indeed, thought he had detected circular muscular fibres in the thoracic duct of man and some animals, and Soemmerring believed in their existence. Mascagni, on the other hand, does not admit them, nor yet Rudolphi. The external membrane, according to Cruveilhier, is formed of elastic yellow tissue (" tissu dartoïde,") to which these vessels owe their power of undergoing great degrees of dilatation, and again recovering their ordinary caliber. Their vital contractility is manifested for many hours after death: thus, if a dog be killed when the process of digestion is in an advanced stage, on opening the abdomen the lacteals, which are found full of chyle, being stimulated by the contact of atmospheric air, contract, and in a few minutes completely empty themselves. But if not examined within twenty-four hours after death this phenomena is no longer displayed; thus showing plainly that it is of vital origin. Again, if we make a ligature on the thoracic duct or any large lymphatic vessel of a living animal, and then puncture it below the ligature, the fluid escapes in jets; whilst, if this be deferred till some hours after death, it comes away slowly and gradually. It is not very easy to make any decisive experiment as to their sensibility in a healthy state, the results being embarrassed in consequence of the severity of the wounds necessarily inflicted in order to reach them, as well as by a doubt as to whether some nervous filaments may not be implicated. But when they are inflamed, as after a punctured wound, or the absorption of any veins, they manifest a high degree of sensibility, and become the seat of intense pain.

The valves seem to be defective or to exist only in a rudimentary state in the minuter ramifications, such as those forming the cutaneous net-works, as injected fluids are here easily made to retrograde and fill the small collateral branches and off-sets; or, perhaps, as M. Breschet suggests, the valves may even here be perfect, but at the same time so delicate as to be easily ruptured by the slightest force, and thus readily allow the mercury to pass. However this may be, regular and complete valves become first manifest in branches of a caliber a degree greater. Their normal form is pouch-like, as in the veins. Those of an annular shape are to be considered as anomalies or instances of incomplete development, inadequate to the complete closure of the vessel, and not as the ordinary configuration, as some recent authors have erroneously asserted. Generally speaking, they are less numerous in the smaller than in the larger vessels or trunks. In the former, their average distance from one another is about an inch. But this varies in different situations: thus they are particularly close to one another in the parietes of the intestines. The frequent narrowings which give to these vessels the appearance of a string of beads seem to be owing, not to the valves, which are obviously farther apart than these contractions, but to the tendency of the mercury to fall into a globular form. The sphincter-like arrangement of fibres represented by Mascagni at the base of the valves has never been found by Breschet; no more than the longitudinal fibres recently described as passing between valve and valve, for the supposed purpose of contracting the intervening spaces, and so forwarding their contents.

In some of the lower animals, the lymphatics are not furnished with valves. This has been found by M. Fohmann to be the case in respect to the small intestines of the lion and several of the other Carnivora. They are also altogether wanting, or at least very slightly developed, in the tortoise and in fishes.

The lymphatic ganglia or glands vary in size from that of a grain of millet up to that of a pigeon's egg; the largest being the mesenteric, bronchial, inguinal; whilst the smaller ones are met with in the carotid canal, on the deep-seated lymphatics of the limbs, in the epiploa, &c. M. Breschet alludes also to their greater softness and relative size in infancy and youth, and their diminution in number and bulk in advanced life. This last fact is explained by M. Lauth as referrible to the general law of diminution of the capillary vessels in old age, and to the circumstance of these glands being, as Meckel and many other anatomists conceive, only a congeries of minute lymphatic vessels.

The existence of glands has not been recognized commonly in the interior of the organs. Certain diseases in various tissues have seemed to give rise to their appearance and development in situations where they had not previously been suspected to exist. Thus in the liver, the spleen, and the brain, lymphatic glands in a morbid condition have been described; but an attentive examination of these pretended glands contained in the midst of masses of diseased substance, has convinced Meckel that frequently, at least, the products of organic changes, quite unconnected with glands, have been mistaken for them.

The lymphatic glands are particularly numerous about those organs which are in relation with substances coming from without; as, for instance, in the neighbourhood of the respiratory and digestive systems.

They are isolated towards the extremity of the limbs, and become more and more numerous as we approach the trunk. They appear to M. Breschet to have no proper enveloping membrane, but a mere thin covering of condensed cellular tissue. They are largely supplied with arteries and veins. He has seen nerves entering them, but whether they merely pass through them, or whether they leave twigs on their way he has not had ocular proof, and other anatomists who have alluded to the subject are equally divided about it.

As to the intimate structure of these glands, M. B. has not much, and nothing very satisfactory to offer; indeed, he attempts little more than to lay before his readers the principal opinions which have been held by previous writers. But, as to whether Malpighi and Mylius, Cruikshank and Werner, were right in admitting the existence of peculiar follicles or roundish cells in their interior, from the soft walls of which the lymphatics take their rise, whilst the blood-vessels are distributed on them, or whether we are to believe with Albinus, Ruysch, Hewson, Meckel, Mascagni, Béclard, and Lauth, that these glands are the mere result of an agglomeration or contortion of the lymphatics themselves, our author has adduced no new facts of a sufficiently decisive nature to satisfy the mind. He conceives that the division into *vasa afferentia* and *vasa efferentia* is uncalled for, the one set being merely a continuation of the other. The pretended vesicles alluded to above are thought by Burdach to be merely the cellular interstices between the convolutions of lymphatics; whilst Meckel and Mascagni have looked upon them simply as the patulous sections of somewhat enlarged ramuscules. Cruveilhier does not enter particularly into the structure of the glands, but expresses his opinion that innumerable inosculations of the minute ramifications take place within them.

Magendie speaks of a peculiar fluid found in the mesenteric glands which escapes on pressure and is most abundant at the centre. It had been mentioned previously by Malpighi, Morgagni, Haller, and others. Bichat compares it to that in the thyroid and thymus glands. Lauth has found it in all the lymphatic glands, and believes it to be contained in the vessels and not in the interstitial portion. Breschet confirms its existence in all, but affirms its greater abundance in those in which it was first observed, namely, the mesenteric. Of its uses we know absolutely nothing.

In the museum at Strasburg, there is an injected preparation which M. Breschet refers to as proving, undoubtedly, that the absorbent glands consist merely of a plexus of lymphatics and connecting cellular membrane; and his own investigations have confirmed him in the belief of there being no proper cavities within them. He admits, however, that the subject is still in need of elucidation.

In the section entitled "Terminations of the Lymphatic System," and which commences with a detailed description of the thoracic duct and a reference to the admirable plates of Meckel published in 1828, M. B. alludes to the fact of its rarely being simple throughout, but on the contrary being for the most part divided into branches which again recombine, and thus make at present a reticulated appearance on a large scale; as also to the circumstance of its being sometimes double in its whole extent;—facts which enable us to explain how its apparent obliteration in one point may be consistent with the continuation of life. This plexiform disposition, occasionally met with in the human subject, recalls the normal condition in certain of the lower classes of animals. Wutzer, Müller, and Bohl, have seen a branch of communication pass from the thoracic duct to the vena azygos; and this, which is a rare anomaly in the human species, is natural in the hog.

After mentioning the termination of the thoracic duct, of the right subclavian trunk of the absorbents, of the right jugular trunk, and left axillary trunk in the subclavian and jugular veins, or at their junction, he alludes to the "vexata questio" as to whether there exist other communications in more remote parts between the lymphatic and venous systems. In some of the lower animals, such connexion has been rendered indubitable by the researches of MM. Fohmann, Lauth, Panizza, and Müller. Thus, for example, the first of these distinguished physiologists has found numerous such communications within the walls of the digestive organs and within the mesentery of several fishes; and Müller has detected inosculations of the lymphatics of the thigh with the sciatic vein in frogs. M. Fohmann has made a similar discovery as to the lymphatics coming from the inferior extremities, and those of the intestines becoming united with the sacral and renal veins in birds. All this, however, is far otherwise with regard to the human subject; and Lippi's pretended discoveries in this respect, namely, communications between the lymphatics of the digestive organs in man with the vena portæ, internal pudic and renal veins, the inferior cava and vena azygos, have been successfully combated by Fohmann, Panizza, and Rossi, who have shewn that the Florentine anatomist has, in his plates and descriptions, obviously mistaken lymphatics for veins, and veins for lymphatics, in a manner little creditable to his caution and accuracy; and when at Paris, it seems, he was quite unable to demonstrate, when requested to do so by M. Breschet, the very things which he had previously figured and described; and Blandin and Cruveilhier have searched in vain for any such communication between these two systems as that spoken of by him. It remains then to consider whether such communication takes place within the lymphatic glands. J. F. Meckel, the elder, adopted the affirmative in opposition to Monro and Mascagni, and it has likewise been supported by Caldani, Werner, and Beclard, and more recently by Fohmann, Tiedemann, Lauth, and Panizza.

It had long ago been observed that mercury injected into the lymphatics entering a gland escaped with as great, if not greater facility, by the veins, as by the efferent vessels. The explanations of this fact have been various; some ascribing it to rupture of the tissue of the gland, others to a natural communication between the two sets of vessels in the interior of the organ, whilst others again look upon it as a simple example of transudation. M. Breschet gives several, and we think adequate, reasons for rejecting the first of these explanations. The second is supported by M. Fohmann, who has not, however, by any means demonstrated the fact; and, were it true, the injection should not only always pass from the lymphatics into the veins, which is far from being the case, but also conversely from the veins into the lymphatics. Now M. Panizza remarks, that, in his numerous injections in regard to the human subject, he has hardly ever known this last to be the case; and from a great variety of experiments in relation to man and other animals, he has come to the general conclusion that in all cases the passage from the veins into the lymphatics is effected, if at all, with considerable difficulty, requiring various precautions, such as tying the neighbouring veins, &c.; that the degree of difficulty varies in different organs; and, lastly, that in those instances where the transmission is effected, we are still quite ignorant as to the mode of it.

The remaining hypothesis suggested by Mascagni, and advocated by Müller and Panizza, namely, that the passage of the mercury from the veins into the lymphatics within the glands takes place by transudation through minute pores, somewhat in the same manner as the air in the air-cells may be supposed to be enabled to act on the blood in the minute vessels of the lungs, appears also to many other modern physiologists to be the most probable explanation of the phenomenon: but we own that none of the hypotheses seem to us quite satisfactory.

The lymphatic vessels have been investigated with much care in fishes by M. Fohmann, and the results at which he has arrived are, that they do not originate by open mouths here any more than in the higher classes, but by culs-de-sac, forming pouch-like dilatations in almost all the tissues of the animal; that their internal surface is polished whilst the external is surrounded by a greater or less quantity of soft spongy cellular tissue, which must be peculiarly favorable to absorption by collecting a quantity of fluid around the walls of the absorbing vessels. This same disposition of parts must be of extreme importance if Treviranus' notion already alluded to prove correct, namely, that the minute ramifications of the lymphatics originate in those elementary cylinders of which the cellular membrane is chiefly made up. The absence of valves in the lymphatics of fish, save just where they enter the veins, as also the want of glands long ago remarked by Monro and Hewson, are singular facts in the comparative anatomy of this system. M. Fohmann, however, thinks that he has discovered rudiments of these latter organs in the neighbourhood of the liver and stomach, and conceives that their spleen, in regard to the great number of lymphatic vessels which it receives, should be looked upon in the light of a great lymphatic gland. The analogy is however rejected by Breschet, and we confess, as it appears to us, with great reason, as it is at once fanciful and unsupported.

After alluding to the easy passage of injection from the uterus into the veins, and vice versâ, as also from these vessels into the excretory ducts of various organs, M. Breschet goes on to mention a somewhat rare case spoken of by Panizza, and which Breschet himself has also met with, namely, the casual injection of the lymphatics on the introduction of mercury into an excretory duct under the pressure of a moderate column of the metal. Thus, for instance, mercury injected into the hepatic duct has been known, without entering either the arteries or veins, to pass into the lymphatics in the transverse fissure of the liver along by the side of the ramifications of the vena portæ, and to fill also those on the convex surface near the entrance of the umbilical vein. A similar transmission, though in a still more limited number of instances, he has occasionally observed to take place on the injection of the lactiferous vessels with mercury. M. Panizza has never succeeded in injecting the lymphatics of the testicle through the vas differens; and as to the mode of communication in those organs where it does take place, he confesses himself totally ignorant. With a view to ascertain whether it might depend on any rupture of the parts concerned, he emptied a biliary vesicle, made many superficial scratches on its internal surface, and introduced them into mercury; but neither by shaking, compression, or any other mode, could he cause it to pass into the lymphatics.

The lymphatic vessels of the intestines have been divided into two orders, viz. the superficial set, or ordinary lymphatics, and the deeper-seated, or chyliferous vessels, or lacteals as they are commonly called; but, as their respective networks communicate freely with one another, it is not always very easy, in point of fact, to distinguish them, and hence some anatomists deny the propriety of the division altogether.

The chyle, as is well known, contains numerous globules. These, according to Leuret and Lassaigne, are round in birds, whilst those in the blood are elliptical; and in the mammifera, instead of being flat, like those of the blood, they are round, as has been determined by the microscopical observations of Müller on the rabbit, the cat, the dog, &c. Like Hewson, he has generally also found them somewhat smaller than those of the blood. The globules found in the lymph, which are much less numerous than those in the blood, must either originate in the decomposition of the organic textures, or be formed within the lymphatics themselves. In the lacteals they have been observed by Müller in the smallest ramifications, although these presented no apertures sufficient for their admission. Their source must be merely matter of conjecture, as we have no sufficient evidence on the subject.

Müller once met with a case which was singularly favorable to investigations into the nature and composition of the lymph, in a young man who had a wound of the instep which had resisted all attempts to heal it, and from which lymph escaped in considerable quantity in jets on pressing with the finger up along the dorsum of the great toe towards the situation of the wound. It had the appearance of a colourless, limpid, inodorous fluid, with a saline taste, turning the vegetable acids slightly green, and coagulating within the space of ten minutes into a reticulated mass. He directed his attention particularly towards determining whether the pure lymph, as distinguished from that in the thoracic duct, which is ordinarily mixed up with chyle, contained globules; the existence of which, notwithstanding the testimony of Hewson has been controverted by Reuss, Soemmering, Tiedemann,' and other modern observers; and the result was that he satisfied himself that it did, in fact, contain a multitude of globules, though of much smaller dimensions and less numerous than those of the blood. During coagulation a portion of these remained in the clot, but the greater part continued in suspension in the serum. It was quite obvious that the globules did not constitute the coagulum, but were merely entangled in and disseminated through it; a discovery of the more interest as it shows a close coincidence with the peculiar theory of the coagulation of the blood advanced by him, and shown to be the true one, in opposition to that of Home, Prevost, and Dumas, and almost all other physiologists.

Müller took the blood of a frog, and, by filtering, separated all the globules, and so obtained a perfectly colourless fluid, in which there formed in the course of a few minutes a transparent coagulum, found to consist of pure fibrine alone. From this it appeared incontestible that the globules had no part in the phenomenon; and this view Berzelius had previously suggested, only, however, in the form of a conjecture, in consequence of discovering fibrine in solution in the lymph of the blood. In the blood of the human subject, the minuteness of the globules throws a difficulty in the way of separating them by filtration; but a similar result has been arrived at in respect to it, by retarding its coagulation till these bodies have had time to subside to the bottom of the fluid; and an examination of the buffy coat of the blood in inflammations would afford additional evidence of the independence of this power of concretion on the globules. The colourless fluid obtained from the blood as described above, resembled perfectly that got from the wound alluded to. Containing all the fibrine, it differs essentially and entirely from the serum, and is called by Müller "the liquor of the blood." M. Breschet is of opinion that this demonstration of the identity of the lymph with one portion of the blood cannot fail to lead to important results in regard to the offices of the lymphatics. M. Dumas thinks that we may form a pretty accurate notion of the lymph by viewing it as blood containing about double the usual quantity of saline matter, diluted with water and then filtered. He supposes that it is actually elaborated from the blood by some process of filtration through the capillaries of the glands, after having been previously loaded with saltish water, introduced into it by endosmosis through the walls of the vessel. Whatever may be thought of this explanation, M. Breschet is satisfied as to the close relationship in chemical character of the lymph to the fluid obtained from the blood in the manner above described.

The development of the lymphatic system, and its differences according to age, are the subject of the eighth section. Of the condition of this system in the fœtal state we may be said to know almost nothing. The attempt of M. Uttini, about thirty years ago, to prove the existence of absorbents in the chord and placenta, appears to have been a total failure; and M. Fohmann's more recent attempts are, as we have already shown, not much more successful.

In the embryo, during the earlier periods of fœtal life, the lymphatic ganglia do not exist; and where they first appear it is under the form of simple plexuses, in which the continuity of the vessels cannot be questioned; a circumstance which adds much probability, as Breschet well remarks, to the essentially plexiform structure of the lymphatic glands.

The comparative anatomy of this system is despatched by our author in a very few pages, and in a manner at once meager and unsatisfactory. Like most other systems, it becomes more and more perfect as we ascend in the scale of animal life. The lymphatics, or vessels specially destined for absorbing and carrying fluid bearing a strong analogy to blood, exist probably only in the vertebrata; at least M. Breschet is very sceptical as to the propriety of bestowing this title, as has been done by M. Carus, on a set of tubes opening on the surface of the body in certain gasteropodous mollusca and some bivalves, and which seem really distended to take up water, convey it to the visceral cavity, and probably extract air from it in its passage; so that they should rather be looked on in the light of organs subservient to respiration.

Our knowledge of the lymphatics of fishes, the existence of which was discovered by Monro and Hewson, has received many recent additions from the labours of M. Fohmann. They form numerous plexuses, and exhibit considerable dilatations between the mucous and muscular, and between the muscular and serous coats of the alimentary canal, and communicate largely and freely. There are no distinct mesenteric glands, although, according to Professor Grant,* "two or three small, lenticular, glandular bodies have been observed in some fishes as high as the œsophagus, which have been regarded as mesenteric glands;" but, according to the same authority, they are considered by Meckel to be analogous to the thymus gland of the superior animals. Neither are there any valves in the lymphatic system of fishes, excepting where the main trunks of the system unite with the veins. In reptiles, mesenteric glands are still wanting, and their place seems to be supplied, as in fishes, by very frequent and complicated anastomoses of lymphatic vessels. It is among reptiles that rudimentary valves begin first to exist. In reptiles and amphibia, the lymphatic system is characterized by the existence of pulsatory vesicles in different parts of the body. These were first discovered by Müller, and very nearly also at the same time by Panizza, who believed that he had also detected them in birds. Müller observed them in the frog, toad, salamander, and green lizard.† In toads and frogs there are two pairs of these vesicles; one situated just under the skin, through which its pulsations are readily seen in the living animal, immediately behind the hip-joint of the posterior extremities; and the other seated more deeply, one vesicle on each side of the body, near the end of the great transverse process of the third cervical vertebra, under the posterior margin of the scapula. The anterior pair receive lymph from the anterior part of the body, and pour it into the jugular vein, the posterior empty their contents into the crural vein. Their pulsations are totally independent of the heart and of the acts of respiration, since they continue after the removal of the heart, and for an hour or two after the apparent death and most complete dismemberment of the animal. Neither are they synchronous with each other on the two sides of the body, nor always performed in the same space of time. It has almost always happened, when we have observed these vesicles, that the pulsations were performed with very little regularity, but had frequent and sometimes long intermissions. Their use is to circulate the lymph through the body, in the same way that the blood is circulated by the heart. Panizza has observed a similar pulsating vesicle in some of the lymphatics of the posterior part of the body, in one of the serpent tribes, (Coluber flavescens;) and a pulsating structure has also been noticed by Dr. Hall‡ in the tail of the common eel, which he designates a caudal heart, and believes to be a portion of the venous system; but there seems more reason to believe that it is a lymphatic vesicle, like those discovered by Müller in reptiles and amphibia, and is perhaps part of some lymphatic structures situated in close approximation with the veins in the tail of the eel. Panizza also observed two lymphatic vesicles, like those in amphibia, in birds. In the goose he found two lymphatic

* Lectures. Lancet, vol. ii. 1834; p. 819.

† Transactions of the Royal Society, Part I. 1833.

‡ On the Circulation of the Blood. 8vo. 1831. Page 170.

plexiform vesicles in the pelvic region of the body, between the tail and the thighs. These are of large size, half an inch in length and one quarter of an inch broad, and, when fluid was thrown into the adjoining lymphatics, it passed not only into the vesicles, but from them into the veins. Müller has recently examined these vesicles, and, according to Dr. Allen Thomson,* has satisfied himself that their contractions and dilatations in the living goose are not dependent on an automatic power of their own, as believed by Panizza, but correspond exactly with the motions of respiration, and cease when respiration is suspended. We are not aware that similar vesicles have yet been discovered in any of the mammalia.

In regard to our knowledge of the lymphatics in birds, much has been added by Tiedemann and Lauth to what was previously discovered by Hunter and Hewson. In this class glands first present themselves, but still few in number; chiefly about the region of the neck and base of the abdominal aorta; and they are, according to M. Tiedemann, most developed in the aquatic tribes. In other parts of the body they are still replaced by plexuses. Fohmann, Lauth, and Panizza, also describe numerous communications between these vessels and the venous system.

It is in the mammifera that the lymphatic system acquires its highest degree of development, the trunks being larger, the valves more complete, the plexuses less numerous, the glands multiplied, and the connexions with the venous system reduced in frequency. They present likewise the new peculiarity of being distributed in two layers, a superficial and a deep seated one, so that they form, as it were, a double system of vessels. They are more developed, or at least larger, in the other mammifera than in man, but their glands are less numerous. In those animals, however, whose intestinal canal is very short, the glands are crowded so close together in the abdomen that they form, as it were, a continuous mass, which has commonly, but improperly, been named the pancreas of Aselli, after their first describer.

Thus, as M. Breschet remarks, in proportion as the organization of the animal becomes more complicated, the lymphatic system reaches higher degrees of perfection, and acquires, so to say, a more independent existence. It gradually loses the cellular and plexiform appearance and assumes the form of distinct canals, its valves become more developed and its glands more frequent.

The physiology of the lymphatic system is discussed in the third chapter. The doctrine of venous absorption, as old as the time of Galen or even of Hippocrates, was vigorously defended in the last century by Swammerdam, Boerhaave, the eldest Meckel, and Monro. The discovery of Pecquet, the forcible arguments of Bartholin and others, aided by the combined powers of truth and time, at length led to the general recognition of the absorbing functions of the lacteals. They were not, however, acknowledged as the exclusive agents in this process, as an intermediate opinion soon sprung up dividing the function between the two sets of vessels, and this view was advocated by Ruysch, Lieberkuhn, Albinus, and Walter, and prevailed till the middle of the eighteenth

* Edinburgh Medical and Surgical Journal, No. 125.

century. It was not, however, destined to remain permanently undisputed. The two Hunters, and especially John, threw much doubt on the fact of the participation of the veins in the absorption of the chyle, and reinstated the hypothesis of the lacteals being the sole agents in the performance of this function. Schreger, Morgagni, and Lauth, followed on the same side, and the question was supposed to have been set at rest when Magendie appeared as the advocate of occasional absorption by the veins. The names of Emmert and Rapp, Meyer, Westrumb, and Foderà, have appeared in support of the same view; and Tiedemann has likewise lent it his efficient aid. Segalas even went farther, asserting that it is the veins alone which absorb foreign substances, such as vegetable poisons, &c. introduced into the intestinal canal.

As to the presence of blood in the lymphatics in any case, M. Breschet is very sceptical, conceiving that the size of the globules of this fluid would form an insurmountable obstacle to their being taken up by them. The affirmative testimony of MM. Foderà and Lauth is however entitled to respect; and, though we may not be able to explain how the blood enters them, we are not therefore justified in rejecting what seems so creditably attested. Thus M. Foderà having put two ligatures on the intestine of a rabbit at a little distance from one another, made an incision near one of the ends, and found shortly afterwards that the lacteals coming from the wound were filled with blood. M. Lauth, again, speaks of a young wolf which having been shot in the breast an effusion of fluid took place into the thorax, and the lymphatics in the neighbourhood were observed to be full of blood, as was likewise the nearer half of each of the glands into which they entered. M. Breschet, however, remarks that as there was no microscopic examination made of the supposed blood, it may have been merely a solution of the colouring matter in a state of decomposition.

The globules of pus being still larger, there must needs be a still greater obstruction thrown in the way of their being absorbed; yet this fluid likewise has frequently been observed within the absorbents. He is inclined to refer both cases if they must be admitted as facts, as we think unquestionably they must be, to some rupture or other abnormal condition of these vessels, and to look upon them not as instances of the rule but rather of the exception.

"To what general conclusion are we then to come," says M. Breschet, "from all these observations, which are in part in harmony with each other and in part so discordant?—There are at least two facts which no one disputes. The first is, that the lymphatics of the mesentery absorb from the intestines filled with the product of digestion, not the chyle itself, but the organic materials of this fluid. They occasionally absorb also foreign substances entire and unchanged, yet always existing either in a state of solution, as certain salts, or of minute subdivision, as is the case with regard to fatty matters. The second fact is, that the lymphatics from all parts of the body bring back towards the heart a fluid of the nature of blood, yet differing from that carried by the arteries and veins in containing none of the red globules. Now since, notwithstanding the employment of high microscopic powers, no communication has hitherto been discovered between their origin and that of the arteries and veins, there is no other conceivable way in which they can get this fluid save by what is commonly understood by the term absorption, a process the effects of which we know, though ignorant of its cause. As to the question whether this absorbing faculty belongs to the lymphatics exclusively, or whether the veins also participate in it, it appears to me less difficult to answer it, than is generally thought. I shall not cite the instances of animals unprovided with a lymphatic system, and in which the process of absorption notwithstanding is equally well performed; for it would be rash to found any conclusion on organizations so remote from that of the vertebrata; nor shall I instance the absorption of certain cerebral effusions, nor that which takes place at different periods of life in the osseous tissue; nor yet that which must undoubtedly alternate with the act of nutrition in the brain, and in the eye; for it is not only possible, but highly probable, that all these parts are furnished with lymphatics; but it seems established by experiment, as strongly as anything can well be, that in man the veins and the lymphatics communicate together in the glands: the only point about which there is any doubt is the mode of this communication. We have every reason, however, to believe that it does *not* resemble that which exists in various parts of the body, in an obvious form, in some other of the vertebrate classes of animals,—birds, reptiles, and fishes,—and which does not differ from the junction of the thoracic duct with the subclavian. It seems if not absolutely demonstrated, at least highly probable, that it is effected neither by direct inosculation, which no one has ever seen, nor yet by patulous orifices at the extremities of the two systems, for these likewise have never been observed by any anatomist: on the contrary, it is perfectly understood that the arterial and venous capillaries are continued into each other without interruption in all regions of the body where minute injections have been made. Consequently, it would seem that it must be by an absorbing power analogous to that ascribed to the lymphatics, that the veins absorb the contents of these latter vessels within the glands. But if probabilities are so strong in favour of this hypothesis in respect to what takes place within the glands, how can we deny to the veins universally a power which they seem so unequivocally to possess in this situation? The only real difficulty consists in determining the limits and circumstances under which the one or the other of these two systems exerts this power, and this the present state of our knowledge is unfortunately inadequate to solve." (P. 216 *et seq.*)

It is a singular fact mentioned by Müller, that the chyle in birds is almost always limpid, whatever be the nature of their food; whereas it is well known that in the mammifera it varies much in relation to the species of ingesta. Thus, in the carnivora it is much more turbid and opaque than in those animals which live on vegetable food.

As to the absorption of gases by the lymphatics of the intestines, admitted by Foderà, Mascagni, and others, M. Breschet rejects it, being, as he thinks, totally at variance with what we know of the fatal effects of the introduction of air, even in very minute quantities, into the circulation. But this, as it seems to us, is getting rid of the matter in too summary a manner. Are there not cases where meteorism of the intestines very suddenly disappears, without any simultaneous disengagement of air externally? and is there not reason to believe that the air in emphysema is occasionally, in part at least, removed by absorption? It does not necessarily happen, as M. Breschet would seem to apprehend, that air or gases, if absorbed, must exist in the circulating fluids in a free or uncombined state. The decomposition of atmospheric air, which takes place within the lungs in the normal state, and the absorption of one of its component parts, is sufficient to prove that aerial fluids are capable of being introduced into the circulation, and existing there, we do not say in a free state, but in some form or other, without producing any of the disturbances alluded to by our author. There is nothing more wonderful in one set of vessels being capable of absorbing certain gases, than in another set secreting the same; and that gases are so secreted into the cavity of the intestines can scarcely be doubted even by the most sceptical.

"Arrived at the extremities of the arterial capillaries, and having lost some of its constituent parts, and especially a portion of its fibrine, the blood is then divided into two parts: all its globules enter into the venous capillaries, whilst the liquid which held them in suspension, and in which the fibrine is dissolved, along with the albumen and the different salts, is returned partly by the veins and partly by the lymphatics. The passage by the veins is comprehended without difficulty; for these vessels are seen to be continuous with the arteries, and very many of them are of sufficient caliber to admit even globules. But, with regard to the lymphatics, it is quite otherwise; so that, in our endeavours to explain such entry of a part of the blood, we are reduced to mere conjecture. We must either suppose, for example, that the arteries allow their contents to transude into the parenchyma of the organs, where the lymphatics take them up; or else that the contained fluid penetrates from one set of vessels into the other through the tissue of their parietes, brought into close proximity with one another. There are some parts of the body where the arteries are reduced to ramifications so minute as no longer to transmit the globules; as, for instance, the ramifications of the central artery of the retina which run towards the posterior part of the capsule of the lens, and which, according to the micrometrical measurements of Treviranus, have a diameter of only 0.0053 to 0.0049 millimetres; whilst the diameter of the globules of the blood is from 0.005 to 0.006 millimetres. Now, it is obvious that the fluid carried by these arteries must return by the venous system; but the veins which carry it back will, in like manner, be too small to convey the globules. There must, then, exist veins also which are colourless, and of such extreme tenuity that we shall in vain attempt to distinguish them from lymphatics." (P. 223.)

This view approaches very close to the opinion held by Dr. Graves, namely, that the veins of the white tissues are identical with the lymphatics.

As to the force which propels the fluid contained within the lymphatics, M. Breschet has no satisfactory theory to offer. This force must evidently be a considerable one, as M. Carus and others have seen the thoracic duct on which a ligature had been tied burst by the mere accumulation of fluid within it, and have observed this fluid in other instances escape in the form of a jet, on making an opening in the side of the vessel. M. Breschet thinks that possibly it may be somewhat analogous to the power which circulates the sap in the vessels of plants, and that the propulsion of the fluid is chiefly determined by the *vis a tergo* or initiatory impulse on its first introduction within the lymphatic capillaries.

According to the experiments of Tiedemann and Gmelin, the thoracic duct is insensible to mechanical and chemical stimulants; and Müller has come to the same conclusion in reference to galvanic influence.

As to the uses of the lymphatic glands, we are still as much in the dark as before M. Breschet favoured the world with the production before us. In regard to the hypothesis of Tiedemann and Gmelin,—namely, that they serve to prepare the red colouring matter of the blood,—M. B. expresses his regret that these able observers had not had recourse to the microscope in confirmation of their views, of the correctness of which he appears very doubtful. His own opinion, however, is of little value on this point, as he does not appear to have bestowed any pains on the investigation of the subject.

M. Breschet's work closes with a chapter on the Pathological Anatomy of the Lymphatic System. This part of his subject is treated of under two heads: 1st. Congenital Malformations, or anormal peculiarities in the form of its vessels and mode of their distribution; 2d. Pathological Lesions. We have not space to follow him through either of these departments, but must remark that the latter, and more practically important of the two, is much more briefly handled than we could have wished, and is very poor in original facts, as well as very incomplete even in the enumeration of already ascertained affections. It must, at the same time, be admitted that our precise knowledge in this department of pathology is still very inconsiderable.

As to the number of lymphatic trunks which go ordinarily to make up the receptaculum chyli, there is an infinite variety of opinions cited by our industrious author; a difference resulting, no doubt, partly from their number being really quite indeterminate, varying ordinarily from three to six; and partly from the superior success of modern anatomists in the injection of these vessels, which has shown them to be generally more numerous than was formerly supposed. The receptaculum itself has been so often sought for in vain in the human subject, that many good authorities have been inclined to discard the term altogether; yet an enlargement at the commencement of the thoracic duct certainly often exists. At the same time, the caution of Portal against mistaking for a dilatation of the vessel itself a thickening caused by its inferior extremity being often surrounded by a number of contorted lymphatics connected by cellular tissue, and enveloped in a cellular sheath, is well founded. Two and even three receptacula have, in some very rare instances, been found, from which as many thoracic ducts arose, and did not coalesce till after they had entered the cavity of the chest. The bifurcations and reunions of the thoracic duct, forming what Haller called "*insulæ*," are so frequent, that Cruikshank thought they constituted the natural state. The subdivisions are sometimes so complicated as to form almost a network.

The division of the thoracic duct at its superior half or superior third is not very rare, and in all such cases one of these branches has been found to terminate in the left subclavian vein, and the other in the right subclavian, or occasionally in the thoracic duct of the right side; which Meckel, the great authority on these subjects, looks upon as a dilatation of the anastomotic branch, which, according to him, passes from the one thoracic duct to the other in the normal condition.

The existence of two thoracic ducts completely isolated and distinct from one another is extremely rare; for they almost always anastomose more or less freely with each other, or are confounded together in some part of their progress.

Under the head of Organic Lesions, the following subjects are treated of:—physical lesions of tissues, including dilatations, and narrowings, and wounds; acute and chronic inflammations of the vessels and glands; tubercular, encephaloid, and melanotic degenerations; morbid growths, osseous, cartilaginous, and fungous; and lastly, alterations in the nature of the lymph itself.

The branches of the lymphatics are sometimes so enormously dilated as to rival the thoracic duct in capacity; and this generally depends on some obvious obstruction in their course, from tumours pressing on them, &c. They sometimes assume a general varicose condition, and at others present a number of partial dilatations, or little tumours, having much the appearance of hydatids. The thoracic duct itself is a still more frequent seat of these partial dilatations than the smaller branches of the system.

The following very interesting case of dilatation of the lymphatics was furnished to our author by M. Amussat. Its diagnosis during life was extremely obscure. This patient, a young man of nineteen years of age, of a good constitution and muscular, died after an illness of only twenty-four hours. About a year previously, a tumour of considerable size had made its appearance in each groin. An attempt had been made to prevent the increase of these tumours by wearing a double truss, which moreover relieved the painful sensation in the part, and enabled him to walk better. Having fatigued himself very much after his arrival at Paris, he was seized with acute pain in the right side of the chest and in the groin; the respiration became difficult, and was accompanied with dry cough, flushed face and headach, fever, and lancinating pain in the tumour. The condition of the patient grew worse and worse rapidly; the tongue, that was at first pale and colourless, gradually became dry; tenderness was felt in the epigastrium, unaccompanied by any tendency to vomiting or hiccup. The bowels were obstinately confined; the breathing was anxious, and dulness of sound on percussion was perceived on the right side below; the tumours were exquisitely sensible and red. A few hours afterwards delirium came on, and the tumours now presented an appearance of fluctuation, the belly became tympanitic and tender all over, and the body livid generally, with extreme prostration. On examination after death, the tumours, which had in some measure collapsed, were seen to be covered with a thin membrane bearing the impression of the pad. On removing this membrane, there was discovered an irregular knotty sac, compared, in regard to its structure, to the vesiculæ seminales, and containing a fœtid puriform fluid. Within the abdominal cavity was found a considerable quantity of bloody serum, but no pus nor any trace of peritonitis. The dissection of the tumour proved that no hernia was present. On the left side, the cyst containing the pus extended down in the crural sheath as far as the inferior third of the thigh. On the right side it passed, in like manner, under the crural arch, but did not extend so far down as on the other side. The peritoneum having been removed on the left side, a collection of pus was discovered, which had made its way as far as the tumours. Pus and a reddish serum were found within the thorax, along with adhesions and congestion of the right lung. On turning over the thoracic viscera, the lymphatics were discovered in a state of obvious disease, being so unnaturally large that there was no difficulty in blowing them up with a pair of common kitchen bellows. The mass of iliac and crural lymphatic vessels was inflated with a straw, and tied, when there was discovered an enormous dilatation of them in the groin; and this it was which had given rise to the appearance of hernia. Another very singular circumstance was observed in this case,—namely, that the iliac glands had entirely disappeared, and seemed to be replaced by the lymphatic vessels. No direct communication with the vein was observed.

In the fourth Number of this Journal will be found a remarkable case of aneurism of the thoracic duct in the region of the solar plexus, by Dr. Albers, of Bonn; and an allusion to another where the receptaculum chyli was found dilated in a dropsical subject. Dr. Albers differs with our author in regard to the effects of the compression of this vessel in the production of dilatation, and remarks that enlarged glands and tumours often press on it without any such effect ensuing; which he is inclined to ascribe partly to the weakness of the current within it, and also to what he assumes to be a fact,—namely, the existence of numerous anastomoses of the thoracic duct with the venous system; a fact, however, of which he adduces no adequate proof. The most frequent cases of dilatation are said to be those in which these vessels contain tuberculous or scrofulous matter.

The lymphatics may be contracted throughout nearly the whole system. Thus Hallé, in looking for the lymphatics of a woman who had died in a complete state of marasmus, in place of these vessels found, in the inguinal regions, nothing but some dryish firm filaments, of a dull white colour, somewhat resembling nervous ramifications; and here and there in their course slight enlargements, the only remaining vestiges of glands.

The effect produced by pressure on the absorbents is often, as we should expect, that they become contracted, obliterated, and finally disappear. Thus, in some cases of aneurism of the aorta, the thoracic duct has been found much diminished in size, or even entirely destroyed.

The importance of the lymphatic system in the production of disease seems, in a part of the last century, to have been considerably overrated, and a number of maladies were referred to it without any sufficient evidence. Thus, rupture of the lymphatics, the very existence of which is hypothetical, was supposed by one writer to be the cause of scrofula in the glands, of tubercle in the lungs, and of tumours in different parts of the body. By others, phthisis, elephantiasis, phlegmasia dolens, and a great number of morbid growths, were referred to the inflammation of these vessels or their glands.

The effects of inflammation on these vessels is best seen in the thoracic duct, which becomes thickened, enlarged, red, and softened in its inner coat, and contains coagulable lymph or pus; and in a more advanced stage may be converted into a fibrous impermeable cord.

Pus has been repeatedly detected, by Dupuytren, Andral, and others, in the lymphatics, leading from abscesses, without any inflammation of these vessels themselves. Hence, where they are mere carriers of this fluid, no change is perceptible in their structure. When, on the contrary, they are themselves inflamed, they present the appearance of a number of red, tense, painful cords; and the cellular membrane around, on making an incision into it, is reddish, dense, and infiltrated with a sanguinolent or puriform serum, or with pus. Having given copious extracts from the work of M. Velpeau on Inflammation of the Lymphatic System, in the fourth Number of this Journal, we do not think it necessary to dwell on the subject again here.

M. Breschet protests against concluding that inflammation of the lymphatic glands is confined to their connecting cellular tissue, from the mere circumstance of its having been found possible to make injections pass through them, when in this state;—for, first, such injection is, in point of fact, often impossible; and, secondly, the lymphatics in other situations, when quite independent of the glands, are known to be occasionally permeable, though obviously and indisputably inflamed.

Tubercular matter has been occasionally found by Cruveilhier and Andral in the lymphatic vessels themselves, as well as in the glands; not, however, by any means so frequently as in these latter. It is not observed with equal frequency in all the glands. Laennec supposed them liable to it in the following order of frequency: the bronchial glands first, then the mediastinal, the cervical, and the mesenteric. But Lombard, from more accurate data,—namely, the dissections of the bodies of one hundred children who laboured under tubercular disease,—found that the different glands were affected in the following proportions: the bronchial in eighty-seven, the mesenteric in thirty-one, the cervical in seven, and the inguinal in three only.

The deposition of tubercular matter, as is now well known, takes place either in the form of infiltration or in masses; the former being more frequent in the bronchial glands, the latter in those of the mesentery.

Melanotic matter has been repeatedly found by M. Breschet, who has written specially on this subject, not only in the glands, but in the lymphatic vessels and in the thoracic duct itself; especially in those cases of melanosis where the disease occupied the mucous membranes, where the tumours were in a state of ramollissement, the matter semifluid, or arranged in layers on the mucous surfaces, or infiltrated into the cellular tissue.

In relation to morbid growths connected with the lymphatic system, M. B. mentions the instances, recorded by Sir Astley Cooper, of its obstruction by means of fungous growths near the lower extremity of the thoracic duct. Ossific deposits are a still more frequent cause of obstruction, both in the vessels and glands. They may take place in the vessels, either as depositions in the substance of their walls, or else in the form of masses within their cavity: the latter appears to be the more frequent. It is, however, chiefly in the glands, and more especially in the bronchial glands, that collections of calcareous matter are met with. Advanced age seems to be one of its predisposing causes, and men appear to be more liable to it than women. Two cases are cited,—one from M. Lauth, and one from M. Andral,—where these deposits co-existed with caries; in one case, of the vertebræ, and in the other, of the ilium; and M. Breschet suspects that the connexion was not merely accidental. Meckel has observed that the development of these ossifications always takes place from without inward, and that there usually remains a cavity in the interior, containing a yellowish substance in layers, bearing a resemblance to tubercular matter, and in close connexion with the osseous granules.

The section which terminates the work is on the Morbid Alterations to which the Lymph is liable. On this hitherto imperfectly investigated subject M. B. throws little new light. The lymph, as we have seen above, is sometimes contaminated by the presence of pus. A case from Lauth is alluded to, in which a sanious fluid was found in the thoracic duct in an individual who laboured under gangrene of the lower extremities. Bile, as has likewise been mentioned, has often been met with in the lymphatics of the liver; and Andral has frequently observed the fluid in the thoracic duct of icteric patients, of a yellowish hue. We have likewise seen that a fluid, like blood, has in a few instances been discovered in the same vessel and in the ramifications of the absorbents; and this is stated on the high authorities of Sabatier, Mascagni, and Soemmering. Notwithstanding the identity of colour of the fluid so found with that of blood, Breschet is inclined to follow the example of Andral, who has prudently avoided pronouncing positively on its nature, bearing in mind the reddish tint which has occasionally been observed in the chyle and lymph by Magendie, in certain circumstances quite unconnected with injury or disease.

M. Breschet closes his work with an interesting case by the elder Sanson, of a man of forty-two years of age, and apparently healthy habit of body, who, in consequence of a slight wound in the right cheek with a blunt razor in shaving, was seized with erysipelatous redness and painful swelling of the face and neck of the same side. In the centre of the swelling on the cheek, and just around the trifling excoriation made by the razor, a few yellowish semi-transparent vesicles were observed on the third or fourth day after the accident; and in the midst of them, and corresponding to the site of the wound, a small irregular-shaped patch, brownish and dried up, was seen, and at first sight mistaken for a "pustule maligne," but, on closer examination, it was found totally free from the emphysematous feeling on pressure characteristic of that affection, and was certainly nothing but the remains of the dried-up excoriation. Being by trade a cabinet-maker, he was not necessarily exposed to any contact with morbid or putrid animal matter, which might have given rise to the affection at first suspected; nor was there any tendency to syncope, irregularity of pulse, or vomiting, epigastric pain, fever, or other general symptoms in evidence of a constitutional affection; and yet the case terminated suddenly and unexpectedly in death on the fifth night after the receipt of the above-mentioned trivial injury. The tumefaction and pain had diminished considerably under the use of simple measures during the few hours he was under M. Sanson's care. A slight difficulty of breathing had been latterly detected, and was attributed by M. Sanson to the probable extension of the tumefaction to the submucous cellular tissue about the glottis. On the evening before his death, he had slight sickness of stomach, which soon ceased of itself, and which was supposed to have been caused by some cherry-water which his wife had imprudently caused him to swallow: the stomach was unaffected, save in this single instance. On dissection, no traces of gangrene nor of putrescency were discovered, with the exception of great liquidity of the blood. The inside of the cheek presented two slight greyish excoriations. The mucous membrane of the stomach was strewed over, in that half next the pylorus, with lenticular pustules, to the number of twenty-five or thirty, depending on tumefaction of the mucous membrane, and covered over with a greyish pseudo-membranous secretion; the rest of this membrane appeared red and inflamed. The small intestines were for the most part healthy, save a few patches, which were somewhat injected, or as it were slightly ecchymosed. The tumefaction of the cellular tissue extended from the cheek and submaxillary region to the neighbourhood of the glottis; and a very slight serous infiltration of the rima glottidis was observed, but apparently quite too inconsiderable to be capable of obstructing the passage of the air. The tumefaction, in all the rest of its extent, depended on the infiltration of a blackish sanguinolent fluid. The neighbouring lymphatic glands were of a dark red colour, and one of them in front of the internal jugular vein, a little larger than natural, appeared to be infiltrated with blood, and contained some small black clots. Most of the mesenteric, lumbar, and iliac glands were in a similar condition, and the lymphatic vessels coming from them were in some instances filled with a black and liquid blood; as was likewise the thoracic duct, in its whole course.

We have thus given a sufficiently full view of all the more important facts and hypotheses contained in M. Breschet's work. It affords, as we have already hinted, a good summary of most of what is known on the subject of the lymphatic system; but whether it is calculated to add to his fame as an original observer, or even as an ingenious theorist, there is much reason to doubt. The highest praise which can conscientiously be conferred on it is, that of furnishing the student with a convenient manual on the anatomy, physiology, and pathology of the absorbent system; and it is for this character that we have chosen it as the subject of analysis. It is not, we think, possessed of that kind of merit which would be likely to obtain for it a permanent station among the standard works on the subject. It displays, as we have already said, evident traces of haste, especially obvious in its defective mode of arrangement, its innumerable and tiresome repetitions, and, above all, the want of decisive experiments and original investigations, by the author himself, bearing on points which have been disputed or hitherto imperfectly examined. The style is very prolix and verbose, and little talent for comprehensive and accurate generalization is anywhere displayed. We cannot, however, conclude without expressing our admiration of the diligence and zeal evinced by a man of M. Breschet's time of life and advanced position in his profession, in thus continuing to give such signal proofs of the active interest which he takes in the present state of those branches of knowledge most nearly connected with his art; and of the zeal and activity with which he strives to keep up with the rapidly advancing current of general science.

---

